# Identification of essential genes in *Coxiella burnetii*

**DOI:** 10.1099/mgen.0.000944

**Published:** 2023-02-01

**Authors:** Georgie Metters, Claudia Hemsley, Isobel Norville, Richard Titball

**Affiliations:** ^1^​ Department of Biosciences, College of Life and Environmental Sciences, University of Exeter, Exeter EX4 4QD, UK; ^2^​ Defence Science and Technology Laboratories, CBR Division, Porton Down, Salisbury SP4 0JQ, UK; ^†^​ Present address: Molecular Microbiology Division, School of Life Sciences, University of Dundee, Dundee, DD1 5AA, UK

**Keywords:** *Coxiella burnetii*, TraDIS, transposon sequencing

## Abstract

*
Coxiella burnetii* is an intracellular pathogen responsible for causing Q fever in humans, a disease with varied presentations ranging from a mild flu-like sickness to a debilitating illness that can result in endocarditis. The intracellular lifestyle of *C. burnetii* is unique, residing in an acidic phagolysosome-like compartment within host cells. An understanding of the core molecular biology of *C. burnetii* will greatly increase our understanding of *C. burnetii* growth, survival and pathogenesis. We used transposon-directed insertion site sequencing (TraDIS) to reveal *C. burnetii* Nine Mile Phase II genes fundamental for growth and *in vitro* survival. Screening a transposon library containing >10 000 unique transposon mutants revealed 512 predicted essential genes. Essential routes of synthesis were identified for the mevalonate pathway, as well as peptidoglycan and biotin synthesis. Some essential genes identified (e.g. predicted type IV secretion system effector genes) are typically considered to be associated with *C. burnetii* virulence, a caveat concerning the axenic media used in the study. Investigation into the conservation of the essential genes identified revealed that 78 % are conserved across all *C. burnetii* strains sequenced to date, which probably play critical functions. This is the first report of a whole genome transposon screen in *C. burnetii* that has been undertaken for the identification of essential genes.

## Data Summary

The DNA sequences generated in this study have been deposited in the NCBI SRA database under the BioProject PRJNA863483, sample accession number: SAMN30049071.

**Impact Statement**

*
Coxiella burnetii* causes Q-fever in animals and in humans, but we know little about the biology of the organism. We used a global mutagenesis method to identify genes which are essential for growth and *in vitro* survival of the bacterium. These newly identified gene products are targets for drugs to treat disease. Our work will now allow others to use our approach to identify virulence genes in *C. burnetii* and this will reveal additional targets for drugs to treat disease, and candidates for inclusion in vaccines.

## Introduction

*Coxiella burnetii* is an intracellular, zoonotic pathogen responsible for causing Q fever in humans. *C. burnetii* is predominantly spread by farmed ruminants such as goats, sheep and cattle [1]. Human infection usually occurs via the inhalation of contaminated aerosols. In humans, Q fever is a disease with varied presentations ranging from a mild flu-like sickness to a debilitating illness that can result in endocarditis [2].

The intracellular lifestyle of *C. burnetii* is unique, with bacteria residing in an acidic *Coxiella *-containing vacuole (CCV) within host cells. CCV formation is characteristic of the normal endocytic pathway, and acidification upon lysosomal fusion – a normally bactericidal defence – stimulates the switch of *C. burnetii* from its small cell variant (a metabolically inactive environmental form) to a metabolically active, replicative large cell variant [3]. *C. burnetii* could not be cultured outside of host cells until the development of an axenic medium, ACCM-2, in 2009 [4]. As a result, little is known about the genetic factors that are responsible for the unusual lifestyle of this organism. A better understanding of essential genes will greatly contribute to the current knowledge of the critical functions of *C. burnetii *.

The *C. burnetii* Nine-mile phase I genome (NMI) reference sequence was published in 2003, revealing a 1 995 275 bp genome with a 37 393 bp QpH1 plasmid, that had been previously sequenced [5, 6]. Genome analysis identified 83 pseudogenes, suggesting ongoing genome reduction [5]. This finding also suggests that a number of genes will not be essential for the lifestyle of *C. burnetii *. Later, the genome sequence of the avirulent laboratory strain *C. burnetii* Nine-mile phase II (NMII) was published, confirming the deletion of a large (~26 kbp) chromosomal segment that results in the formation of a truncated lipopolysaccharide without O-antigen sugars [7–9].

To date, there are 83 *C *. *burnetii* sequences deposited in the NCBI database. These sequenced *C. burnetii* strains each fall in to one of six genomic groups (GGs), showing significant genetic diversity amongst strains which have been associated with different hosts or regions located, and show different virulence presentations in animal models [10]. GG I isolates have a global distribution, and include the NMI and NMII strains commonly used in research [11, 12]. GG II isolates are generally European, and contain strains responsible for a large Q fever outbreak in the Netherlands [13]. Recently, GG II has been divided into three subgroups (GG IIa, GG IIb and GG IIc) based on genome content and SNP profile [13, 14]. GG III isolates are associated with cattle [15], whereas GG IV isolates are associated with goats, and do not grow in axenic media [15–17]; GG IV has also been divided into three subgroups (GG IVa, GG IVb and GG X) [14]. GG V isolates are found in North America [12], and GG VI (Dugway) isolates are associated with rodents, and do not appear to cause human disease [18, 19].

Although the first genetic manipulation of *C. burnetii* was performed in 1996 [20], a suitable suicide plasmid system for transposon mutagenesis was not created until 2009 [21]. Since then, transposon mutagenesis has been used to identify many genes encoding proteins that play important roles in *C. burnetii* infection. For example, transposon insertion in *ftsZ* resulted in incomplete cell division, resulting in a significant growth defect in Vero cells [21], and transposon insertion into *CBU_1260* (OmpA) resulted in a defect in host cell invasion [22].

In 2009, four research groups published methods combining transposon mutagenesis with high-throughput sequencing [Tn-seq, TraDIS (transposon-directed insertion site sequencing), HITs and IN-seq] [23–26]. A large population of tens, or hundreds of thousands of transposon mutants can be pooled together, put under selective conditions and simultaneously assayed to identify genes essential for growth and survival in that condition. A range of conditions can be applied to identify (for example) genes essential for infection-relevant conditions (virulence-associated genes), environmental survival and stress response. In addition, investigation of essential genes conserved in the core genome of a species allows the identification of genes which probably play critical functions.

The aim of this work was to generate a transposon library for *C. burnetii* to permit the identification of essential genes, thereby providing information on *C. burnetii* genes fundamental for the growth and survival in ACCM-2 and enabling further analysis of essential gene products as prospective drugable targets. Furthermore, conservation of essential genes in the core genome of *C. burnetii* has been determined. Application of this high-throughput mutagenesis technique has not been previously carried out for *C. burnetii* and will greatly increase our knowledge and understanding of a pathogen for which genetic manipulation techniques have only recently become available. This study provides important new information about the biology of this organism and opens opportunities to study gene fitness in a range of biologically relevant conditions. In the longer term it could lead to new pre-treatments and therapies for Q-fever.

## Methods

### Bacterial strains and culture conditions

*C. burnetii* Nine Mile Phase II (NMII) strain RSA493 clone 4 [27] was grown in laboratory-prepared ACCM-2 [4] at 37 °C in microaerobic conditions (5 % CO_2_, 2.5 % O_2_) for 7 days for liquid cultures, unless otherwise stated. To obtain colonies, bacteria were plated onto ACCM-2 plates containing agarose at a final concentration of 0.25 % (w/v) and incubated at 37 °C in microaerobic conditions (5 % CO_2_, 2.5 % O_2_) for 14 days. *Escherichia coli* strain DH5α was used for replication of the pKM225 plasmid (accession no. HQ386859) and grown overnight at 37 °C with agitation. Where appropriate, antibiotics were added to media as detailed in methods below.

All manipulations of *C. burnetii* NMII were carried out in a class I microbiological safety cabinet in a Biological Safety Level 2 (BSL2) laboratory.

### Generation of *C. burnetii* transposon mutants

pKM225 was introduced into stationary phase *C. burnetii* NMII via electroporation. *C. burnetii* was made electrocompetent through washing with ice-cold, sterile filtered 10 % (v/v) glycerol prior to electroporation of 50 µl aliquots at 1.8 kV, 500 Ω and 25 µF using 0.1 cm cuvettes and an ECM630 electroporator (BTX Harvard Apparatus). Immediately after electroporation, 950 µl RPMI-glutamax was added to cuvettes. Five hundred microlitres of sample was used to inoculate 6 ml ACCM-2 medium in duplicate. Cultures were incubated overnight, before the addition of chloramphenicol at 3 µg ml^−1^, followed by a further 4 days of incubation. To obtain colonies, samples were plated onto ACCM-2 agarose plates containing chloramphenicol at 3 µg ml^−1^. After 14 days of incubation, colonies were washed from the plate and pooled. One hundred microlitres was used to inoculate triplicate 25 ml ACCM-2 cultures for TraDIS library preparation.

### Genomic DNA extraction

Genomic DNA (gDNA) was extracted from 25 ml 7 day old cultures using the GenElute Bacterial Genomic DNA Extraction kit (Sigma-Aldrich) following the protocol for Gram-positive bacteria with slight modification. Cultures were centrifuged for 15 min at 4 000 r.p.m. and resuspended in 760 µl Gram-positive lysis solution before separating into 4×190 µl aliquots to process in four columns. The protocol was then followed with an extended 16 h step in lysis solution and addition of proteinase K. DNA was concentrated by ethanol precipitation and pooled for each 25 ml culture.

### TraDIS library preparation

Unless otherwise stated, the manufacturer’s instructions were followed for all kits in this process. Five micrograms of gDNA was fragmented by ultra-sonication in a COVARIS E220 focused-ultrasonicator at a peak incident power of 140 W for 55 s. Small (<150 nt) fragments of gDNA were removed using the GeneRead Size Selection Kit (Qiagen). gDNA fragments were end-repaired using the NEBNext DNA Library Prep Master Mix Set for Illumina (New England Biolabs) and cleaned up using the QIAquick PCR purification kit (Qiagen). End-repaired samples were A-tailed using the NEBNext DNA Library Prep Master Mix Set for Illumina (New England Biolabs) followed by a further clean up using the MinElute PCR purification kit (Qiagen). Adapter ligation was performed by combining end-repaired, A-tailed gDNA with phosphorylated, annealed adapters and 5 µl ligase and incubating at room temperature for 15 min (for adapter sequences see Table S1, available in the online version of this article). Adapter ligated samples were cleaned up using the MinElute PCR purification kit (Qiagen). Parallel PCR was performed by mixing 10 µl adapter-ligated DNA with 50 µl NEBNext Q5 Hot Start HiFi PCR Master Mix (New England Biolabs), 39 µl nuclease-free water and 0.5 µl each of 100 µM primers Himar-PCR-3 and one of three custom Illumina primers MPX1–3 (for primer sequences see Table S1). Each sample was separated into 2×50 µl aliquots and PCR was performed on the following thermal profile: 2 min at 98 °C, followed by 20 cycles of 10 s at 98 °C, 30 s at 57 °C and 30 s at 65 °C, followed by 5 min at 65 °C. At the end of the reaction, aliquots were re-pooled and PCR contaminants removed with a two column clean-up using the Generead Size Selection kit (Qiagen). Size selection was performed by electrophoresis of a 2 % (w/v) agarose gel. Fragments (350–500 nt) were excised and DNA was extracted using the MinElute Gel Extraction kit (Qiagen). Samples were diluted to 2 nM and submitted for sequencing as 150 bp single-end reads on an Illumina MiSeq platform (Exeter Sequencing Service).

### TraDIS data analysis

Sequencing data were analysed using the Whiteley Lab pipeline [28–30] (available at: www.github.com/WhiteleyLab/TnSeq). First, reads were screened to identify the TGTTA transposon tag, allowing 0 mismatches. The transposon tag was trimmed, and resulting reads mapped to the *C. burnetii* RSA493 reference genome with bowtie2 (v2.4.5) by implementing the TnSeq2.sh script. A correction for polymerase slippage was performed by taking reads that map within 1 bp of each other and collapsing them to the local maxima using slippage.sh script [29, 31]. Next, the TnSeqDESeq2Essential_mariner.R script was executed. This analysis uses estimateSizeFactors() from DESeq2 (v1.16.0) to normalize samples for sequencing depth before generating pseudodatasets containing the same number of insertion sites, and corresponding number of mapped reads as the inputted experimental dataset, randomly distributed across the genome at TA sites. One thousand pseudodatasets were created. A custom .gff file was created in Microsoft Excel to trim 10 % of the 3′ end of each gene, before insertion sites were binned to obtain gene-level insertion counts. DESeq2 (v1.16.0) was used to compare expected insertions per gene (from the pseudodatasets) to observed insertions per gene (from the experimental datasets). Genes were classified as essential if they had a *P*_adj_ value of <0.05, were classified as ‘reduced’ by mClust analysis, and had an uncertainty of <0.05.

### Further bioinformatic analyses

Essential genes were compared to the core genome of *C. burnetii,* as well as the core genomes of different *C. burnetii* genomic groups, using the pan-genome analysis pipeline BPGA (https://sourceforge.net/projects/bpgatool/) as described previously [14]. Clusters of orthologous groups (COGs) were assigned to essential genes using Eggnog Mapper (v2) (http://eggnog-mapper.embl.de/). Proteins encoded by *C. burnetii* essential genes were investigated by BlastP similarity against the Database of Essential genes (DEG) [32].

## Results and discussion

### Transposon mutagenesis of *C. burnetii*

Transposon insertion libraries were generated in *C. burnetii* NMII using a *Himar1* Mariner transposon which inserts at TA sites in the genome, harboured on the plasmid vector pKM225. As pKM225 cannot replicate in *C. burnetii,* transposition results in the loss of the transposase, thus creating transposon mutants with a single, stable insertion [33]. Transposon mutagenesis yielded approximately 3000 colonies per electroporation. Mock electroporation reactions, using nuclease-free water in place of pKM225, resulted in 20 times fewer colonies. These probably represent spontaneous chloramphenicol-resistant mutants, which should not be amplified during subsequent library preparation stages. From six individual electroporation reactions, approximately 20 000 colonies were picked from ACCM-2 agarose plates and pooled to form the TraDIS library.

### Identification of essential genes

The Whiteley lab pipeline is a custom Unix, Perl and R pipeline that has been implemented in previous transposon sequencing studies [28, 29, 31]. The initial step – using the TnSeq2.sh script – was to search reads for the TGTTA transposon tag, with 0 mismatches allowed. The tag is subsequently trimmed and the reads mapped to the *C. burnetii* RSA493 NMI reference genome using bowtie2 with one mismatch allowed. The use of the NMI reference genome as opposed to the NMII genome sequence permitted a quality check that the mapping of insertion sites was accurate, as it allowed the visualization of the absence of reads mapping to the region from *CBU_0679* to *CBU_0698*, due to the deletion of the lipopolysaccharide (LPS) synthesis cluster in strain RSA439 (NMII) compared to RSA493 (NMI).

From a total of 22 776 700 sequencing reads, 21 358 299 (94 %) started with the transposon tag. Of these, 17 826 711 (83 %) reads were successfully mapped to the reference genome. Mapping identified 10 129 unique insertion sites, amounting to one insertion every ~200 bp. The total number of unique insertions disrupts approximately 8 % of available TA sites. The TGTTA sequence occurs naturally at 1851 positions in the *C. burnetii* genome, with 1846 located within coding sequences. It is possible that this could result in false identification of up to 1846 insertion sites. Another potential bias arises from the use of *Himar1* which has shown reduced insertion into the sequence [CG]GNTANC[CG] [34]. As expected, *Himar1* insertions into this sequence were not observed.

**Fig. 1.**
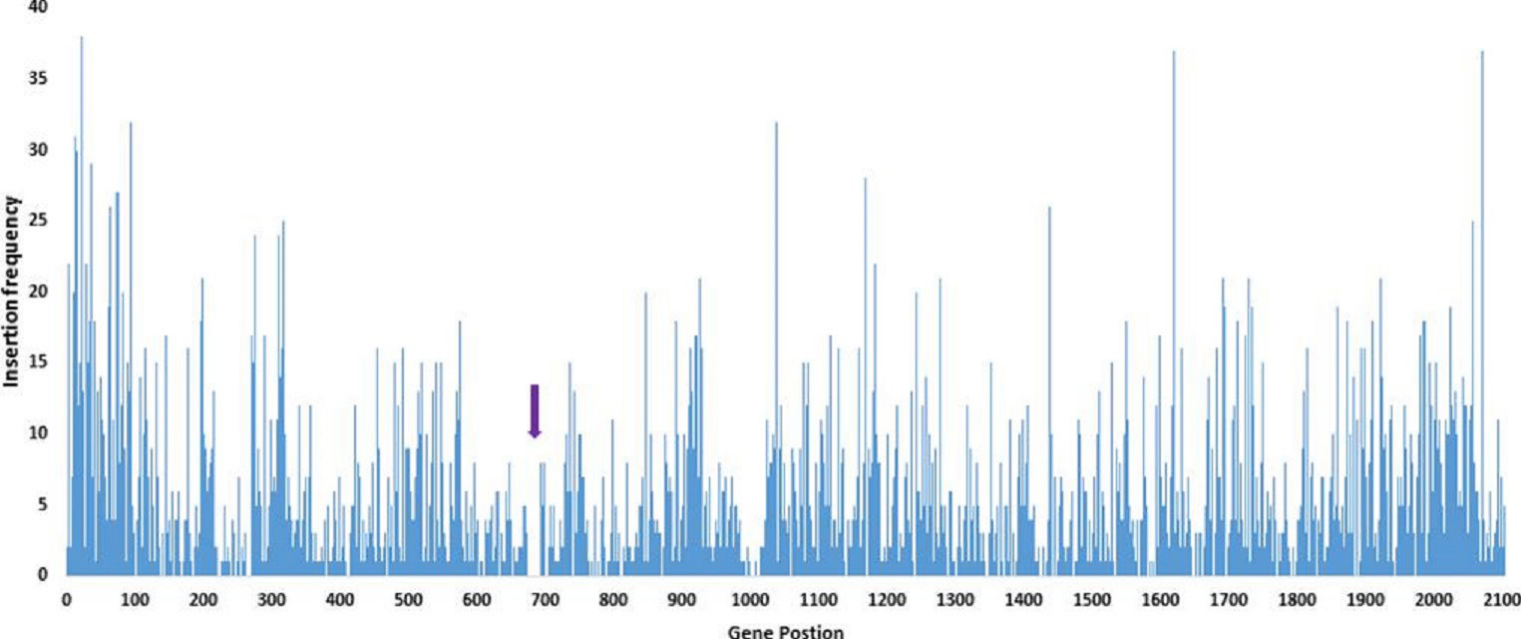
Insertion site distribution across the genome as determined by the Whiteley lab pipeline. The *x*-axis shows gene position, and *y*-axis shows insertion frequency. Regions with no insertions are possible essential regions, with the exception of position *CBU_0679* to *CBU_0698* (purple arrow) which shows the expected LPS deletion in NMII.

The *C. burnetii* genome contains 2134 coding sequences [5]. Up to 38 unique insertion sites per gene occurred, with a mean of four. Visualization of the insertion counts across the genome showed that 71 %(1514/2134) of genes had been disrupted (Fig. 1). These data represent the ‘observed dataset’.

**Fig. 2.**
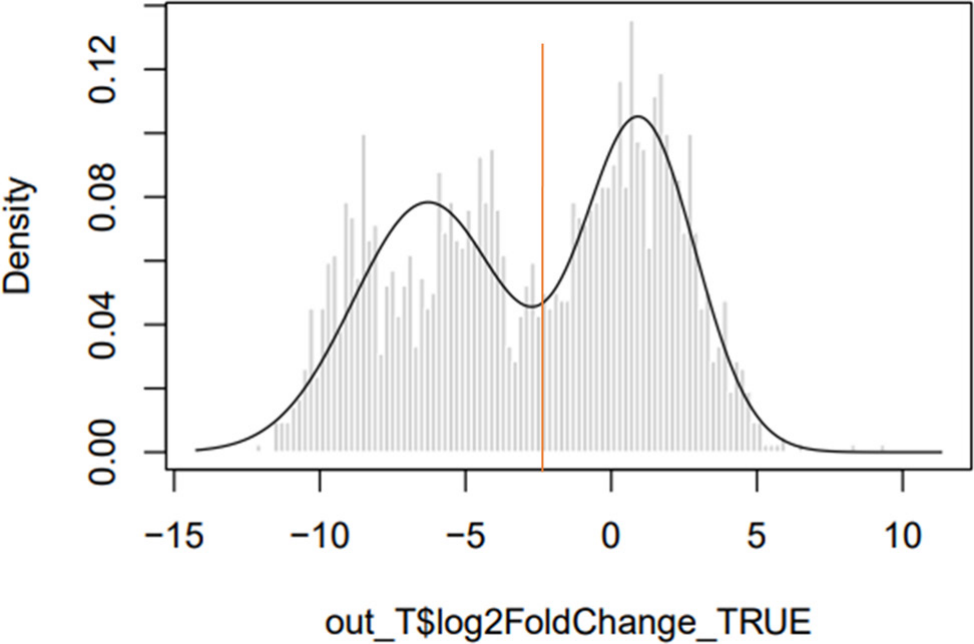
Density histogram showing the log fold change of expected vs observed insertion counts. The *x*-axis shows the log fold change between observed and expected datasets, and the *y*-axis shows the density. The left-hand peak contains genes with fewer insertions than expected, while the right-hand peak contains genes with an expected, or greater than expected number of insertion sites. The red line indicates the log_2_ fold change cut-off of −2.3; genes that fall on the left-hand side of this line are considered to contribute to the left-hand ‘reduced’ peak.

Pseudodatasets were generated by arranging the ~10 000 insertions and the corresponding number of reads randomly at TA sites on the *C. burnetii* RSA493 NMI genome. This simulation was carried out 1000 times, although no significant difference was seen above 500 pseudodatasets. This represents the ‘expected dataset’. Finally, DESeq2 was used to determine whether insertion site abundance in the observed dataset was significantly different from the expected dataset. When plotting a histogram of the log fold change between observed insertions per gene vs expected insertions per gene (Fig. 2) a bimodal ‘line of best fit’ is fitted by the mClust R package, which also determines a log_2_ fold change cut-off value for separation of the two peaks, and genes with fewer insertions than expected are found in the left-hand peak, and genes with the same or more insertions than expected are found in the right-hand peak. Genes were classified as essential if they contributed to the left-hand side ‘reduced’ peak (log_2_ fold change <−2.3), with an uncertainty of <0.05 and an adjusted *P* value (*P*adj) <0.05. It should be noted that although a bimodal distribution has been fitted by the mClust algorithm incorporated into the pipeline, the dataset appears to show a trimodal distribution with two possible groups of genes included in the essential dataset. To determine whether the cut-off value selected by the pipeline was correct, we searched for homologues in the database of essential genes in the two groups [32]. When a more stringent cut-off of log_2_ fold change <−7, we found that 76.6 % (271/354) of the genes were homologues. Using the default cut-off of log_2_ fold change <−2.3 we found that 76.8 % (358/466) of the genes were homologues. The more stringent cut-off excluded genes known to be essential for fundamental biological processes, such as *dnaA*. Together, this suggests that essential genes are found at similar frequencies across both peaks. It should also be noted that there is a valley between the two peaks. Unlike other available pipelines such as BioTraDIS, the Whiteley lab pipeline does not classify any genes as ambiguous, i.e. falling close to the cut-off. This may result in the incorrect classification of genes with log_2_ fold changes close to the cut-off.

Based on the above criteria, the Whiteley lab pipeline revealed 505 predicted essential genes within the chromosome, and six predicted essential genes on the QpH1 plasmid. The *C. burnetii* essential genome contains 24 % of the total genes. This is in line with studies of gene essentiality in other microorganisms, where the essential genome can range from 5 to 35 % of the total genome content [35–39]. Notably, the list of essential genes generated with the Whiteley lab pipeine contains genes that have been shown to be essential for fundamental biological processes, such as DNA supercoiling-associated genes *gyrAB* and all 22 tRNA synthetases.

There have been numerous studies that isolated large numbers of *C. burnetii* NMII transposon mutants for individual mutant screening [22, 33, 40, 41] which obtained transposon insertions in 55 of the essential genes identified in this study. It is possible that the classification of these genes as essential could be false positives. Six of the genes where transposon insertion mutants had previously been reported in other studies had insertions in the 3′ end of the gene, potentially allowing the production of a truncated, but functional product. Single transposon mutants have been reported for the majority of the remaining 45 genes, and it is possible that the mutations could be mismapped. However, to reduce the possibility of false positives in our dataset, 45 of the genes in which others have obtained transposon insertions that are not at the extreme ends have been removed from the dataset. This left a total of 466 essential genes (Table S2).

### Assignment of essential genes to clusters of orthologous groups

**Fig. 3.**
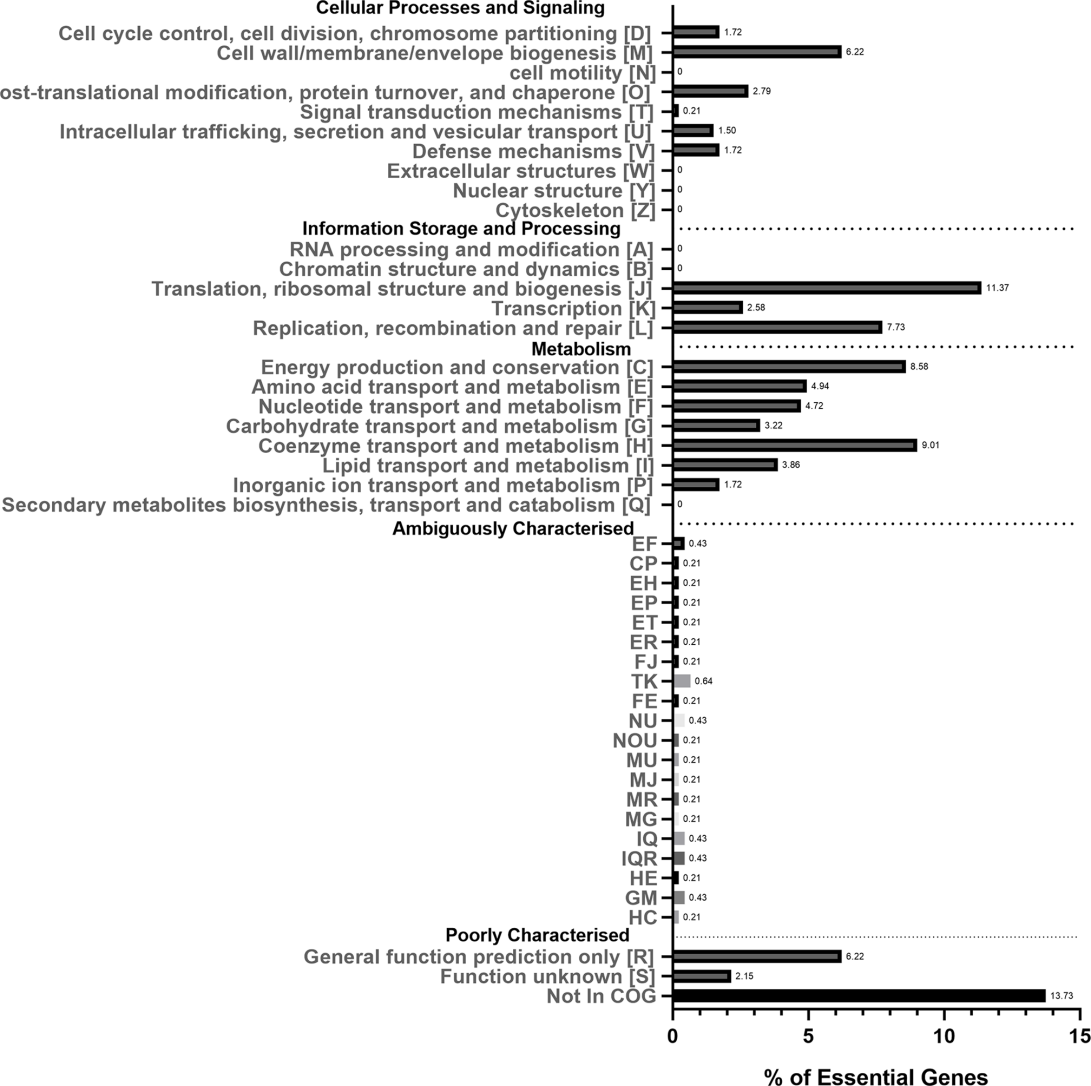
COG classification of essential genes. The *x*-axis shows the percentage of essential genes, and the *y*-axis shows COG categories. Ambiguously characterized groups show the percentage of essential genes that have been assigned to more than one COG category. The numbers next to the bars show the percentage of essential genes for that category.

Essential genes were assigned to COGs [42] to give insight into the dominant essential gene functions (Fig. 3). The COG categories most highly enriched for essential genes were category J (Translation, ribosomal structure and biogenesis; 11.3 %), category H (Coenzyme transport and metabolism; 9.0 %), category C (Energy production and conservation; 8.6 %) and category L (Replication, recombination and repair; 7.7 %). Moderately enriched COG categories were category M (Cell wall and outer membrane structure and biogenesis; 6.2 %), category E (Amino acid transport and metabolism; 4.9 %) and category F (Nucleotide transport and metabolism; 4.7 %). Genes that are poorly characterized or have no COG annotation account for 22 % of the essential gene set of *C. burnetii,* indicating that further trends between bacterial species may emerge as genome annotations improve. These findings are in agreement with other essential gene studies. A comparison of essential gene features between 15 bacterial and one archaeal species [43] found that the COG J – Translation, ribosomal structure and biogenesis was overrepresented in all 16 species discussed, and H – Coenzyme transport and metabolism were over-represented in 87 % of species.

### The *C. burnetii* essential genome is similar to that of other bacterial species

Protein sequences of the 463 essential genes identified in this study were searched against the database of essential genes [32] by BlastP using a cut-off E value of ≤1×10^−10^ for hits to be considered matches. This resulted in hits for 77 % (358/463) of essential proteins against proteins within the database of essential genes. Of the proteins without hits, 47 % (51/108) were hypothetical proteins, explaining their absence in the database.

In the database of essential genes, genes encoding hypothetical proteins comprise 0–48 % of the essential genome of different species, with an average of 14 %. In the present study, 13 % (62/463) of the *C. burnetii* essential genes encode hypothetical proteins. Further elucidation of the structure and function of the proteins encoded by these genes may reveal important aspects related to the physiology of *C. burnetii* that allows it to survive and grow in the acidified endosome.

In addition, the essential gene dataset was compared to a list of 20 universally conserved essential COGs determined by Grazziotin *et al.* (Table 1) [44]. All of the 20 universally conserved essential COGs were represented in the list of *C. burnetii* essential genes.

**Table 1.** A list of universally conserved essential COGs proposed by Grazziotin *et al.* which were also identified in this study

*Information storage and processing*					
*Translation, ribosomal structure and biogenesis (J)*	*CBU_2008*	COG0018		Arginyl-tRNA synthetase	
	*CBU_0205* *CBU_1488*	COG0008		Glutamyl- and glutaminyl-tRNA synthetases	
	*CBU_1248*	COG0124		Histidyl-tRNA synthetase	
	*CBU_0559*	COG0495		Leucyl-tRNA synthetase	
	*CBU_0081*	COG0442		Prolyl-tRNA synthetase	
	*CBU_1188*	COG0172		Seryl-tRNA synthetase	
	*CBU_0241*	COG0090		Ribosomal protein L2	
	*CBU_0238*	COG0087		Ribosomal protein L3	
	*CBU_0239*	COG0088		Ribosomal protein L4	
	*CBU_0253*	COG0097		Ribosomal protein L6P/L9E	
	*CBU_1748*	COG0102		Ribosomal protein L13	
	*CBU_0244*	COG0092		Ribosomal protein S3	
	*CBU_0262*	COG0522		Ribosomal protein S4 and related proteins	
	*CBU_0255*	COG0098		Ribosomal protein S5	
*Transcription (K)*	*CBU_0263*	COG0202		DNA-directed RNA polymerase, alpha subunit/40 kDa subunit	
*Replication, recombination and repair (L)*	*CBU_0659*	COG0592		DNA polymerase III sliding clamp (beta) subunit	
*Cell cycle control, cell division, chromosome partitioning (D)*	*CBU_0562* *CBU_0770* *CBU_1027* *CBU_1580*	COG0037		Predicted ATPase of the PP-loop superfamily implicated in cell cycle control	
*Intracellular trafficking, secretion, and vesicular transport (U)*	*CBU_0258*	COG0201		Preprotein translocase subunit SecY	
	*CBU_1087*	COG0552		Signal recognition particle GTPase	
*Nucleotide transport and metabolism (F)*	*CBU_1830*	COG0462		Phosphoribosylpyrophosphate synthetase	
					

### *C. burnetii* essential genes are highly conserved in GG-I

Previous studies comparing different strains of *C. burnetii* have identified a core genome that is conserved between all strains, consisting of 1311 genes [14]. We found that only 67 % (310/463) of essential genes were conserved across all strains, highlighting the genetic diversity of *C. burnetii* and indicating that different genomovars may have different repertoires of essential genes. This is unsurprising as the *C. burnetii* core genome is derived from a range of *C. burnetii* strains, including those in GG IV that grow poorly (or do not grow) in ACCM-2 medium [16, 45] and the Dugway strains of GG VI, which have a much larger genome and which appear to be avirulent to humans and in immunocompetent animal models of *C. burnetii* infection [46–48]. Future work investigating the suitability of essential genes as novel drug targets to protect against *C. burnetii* infection should focus on essential genes found in the core genome, or essential genes conserved in genomic groups associated with disease in humans or animals.

Next, the conservation of essential genes between different genomovars was investigated. The core genomes of isolates assigned to GG I, IIa, IIb, III, IV, V or VI contain 1810, 1781, 1702, 1875, 1573, 1844 or 1978 genes respectively [14]. The highest number of conservation of essential genes (430/464, i.e. 92 %) was found in isolates assigned to GG I (including the NMII strain), for which the ACCM-2 culture medium used in this study was designed. In contrast, the lowest number of the essential genes (361/463, i.e. 77 %) was found in available isolates assigned to GG IV, which contains isolates shown to be unable to grow in ACCM-2 [16, 17]. The core genomes of GG IIa and GG IIb, which contain the strains associated with the Netherlands outbreak, had a similar number of conserved essential genes as GG I (426/463, i.e. 92 %; and 421/463, i.e. 91 % respectively), as did the cattle-associated isolates of GG III (423/463, i.e. 91 %). Finally, 403/463 (i.e. 87 %) of essential genes are conserved in GG V, the north American strains, and 419/463 (i.e. 90 %) of essential genes are conserved in GG VI, the strains that do not cause disease in humans.

### Essential genes involved in important *C. burnetii* pathways

**Fig. 4.**
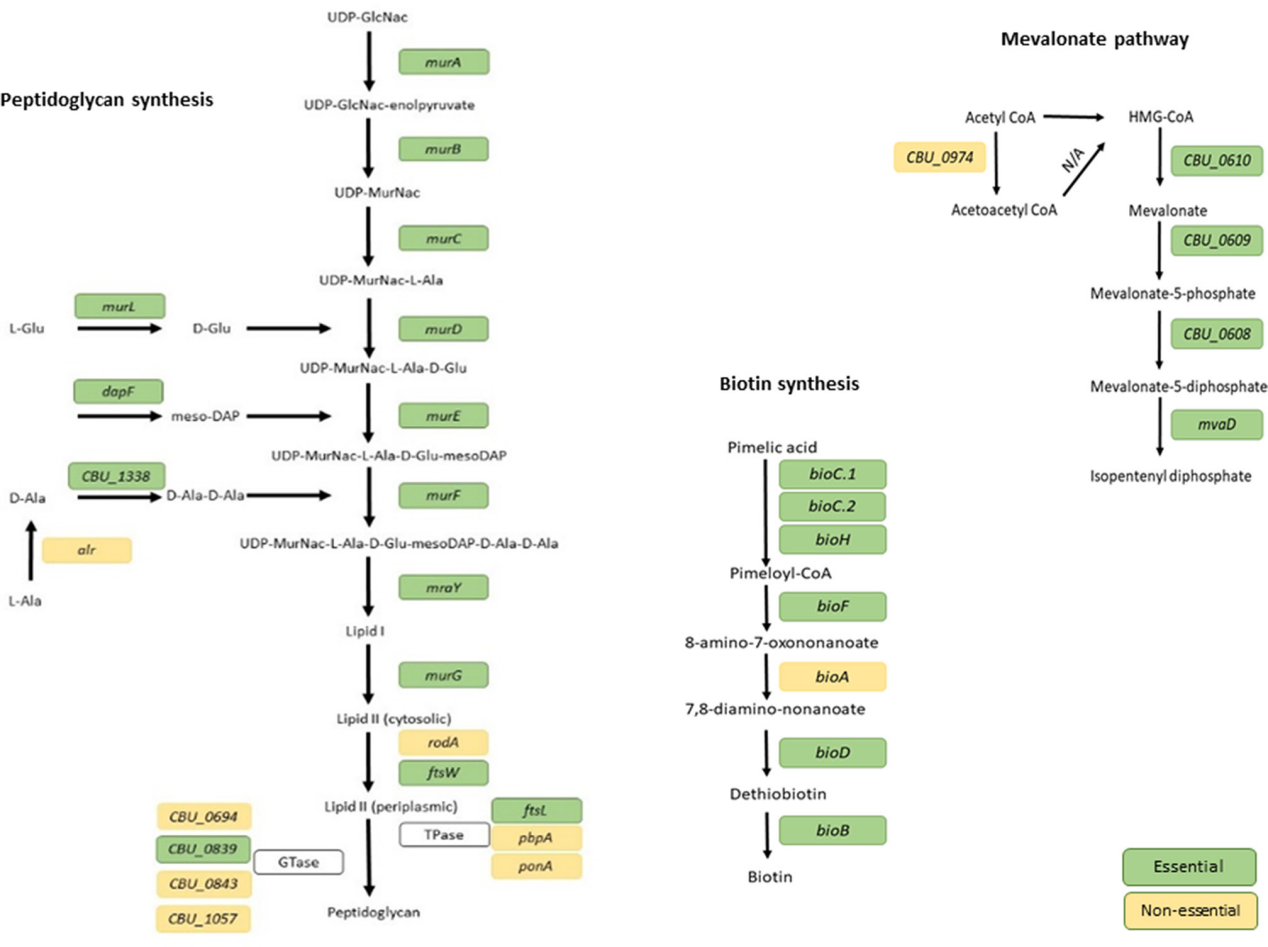
Essential pathways of *C. burnetii.* Essential routes to synthesis were identified for peptidoglycan and biotin, as well as the mevalonate pathway. Essential genes are depicted in green while non-essential genes are depicted in yellow.

Peptidoglycan plays a key role in maintaining cell wall structure, and the majority of the genes in the peptidoglycan biosynthesis pathway were found to be essential in *C. burnetii *. Most genes in the mevalonate pathway were also classified as essential in this study (Fig. 4). Unlike most Gram-negative bacterial species, which source isoprenoids and isopentenyl diphosphate through the glyceraldehyde-3-phosphate pyruvate pathway [5], *C. burnetii* utilizes the mevalonate pathway to source isoprenoids and isopentenyl diphosphate [49, 50]. In previous work, significant upregulation of genes in the mevalonate pathway was seen during infection of *Galleria mellonella*, mice or Buffalo Green Monkey (BGM) cells with *C. burnetii* [51]. In *Staphylococcus aureus,* downregulation of the mevalonate pathway results in the widespread downregulation of metabolism-associated genes, and the upregulation of virulence factors and cell wall biosynthesis genes [52]. In addition, the mevalonate pathway has been shown to play a role in virulence of *Listeria monocytogenes* [53], *Mycobacterium tuberculosis* and *Klebsiella pneumoniae* [54–58]. Many genes in the biotin synthesis pathway and the transcriptional repressor of the biotin operon *birA* were identified as essential genes (Fig. 4). Biotin synthesis has been shown to be critical to the virulence of other intracellular pathogens such as *Francisella tularensis* [59–61] and *M. tuberculosis* [62, 63]. The essentiality of biotin synthesis in these species has been attributed to the absence of *bioP* and/or *bioY* in their genomes, high-affinity biotin transporters which *C. burnetii* is also lacking. As a result, these pathogens require high levels of exogenous biotin, compared to species which possess *bioP* and/or *bioY* [64]. The identification of genes typically associated with virulence in this essential gene screen may be due to the media used. In other bacterial species, TraDIS studies are typically carried out after growing bacteria in rich media. In contrast, ACCM-2 growth medium was developed to mimic the acidic *Coxiella *-containing vacuole that *C. burnetii* resides in within host cells [4]. Therefore, it may not be surprising that some virulence-associated genes have been identified in our study.

### Essential genes involved in axenic growth of *C. burnetii*

*C. burnetii* requires microaerobic conditions for optimal growth, as demonstrated by the expression of cytochrome *bd* (encoded by *cydAB*) [4]. Although *cydB*, encoding subunit II of cytochrome D, was identified as essential, *cydA.1* and *cydA.2* encoding subunit I were not. It may be that *cydB* encodes an essential subunit of cytochrome D. A similar finding was made in some strains of *M. tuberculosis,* where only the *cydB* subunit was classified as essential [65, 66]. However, although *M. tuberculosis* can occupy low oxygen environments [67], unlike *C. burnetii* it is not a strictly microaerobic bacterium. It is also possible that *cydB* has an essential role in protection against oxidative agents in the phagolysosome, as *cydB* mutants in *E. coli* and *Brucella abortus* show hypersensitivity to hydrogen peroxide [68, 69].

*C. burnetii* requires high levels of l-cysteine for axenic growth [4], and *csdB* (also known as *sufS*; the gene conferring a group II cysteine desulfurase) was essential in our study. Cysteine desulferases catalyse the decomposition of l-cysteine to l-alanine and sulfane sulphur, with the aid of pyridoxal 5′ phosphate as a cofactor. This process provides precursors to many biosynthetic pathways such as iron–sulphur (Fe-S) cluster assembly and the synthesis of biotin, lipocic acid, thiamine and nitrogenase [70]. Generally, *csdB* is co-expressed with five additional genes, *sufA, sufB, sufC, sufD* and *sufE* [71, 72]. *sufBCD* were classified as essential in this study. The proteins encoded by these genes form a complex for Fe-S biosynthesis [73], which has been shown to be essential under oxidative stress and iron limitation [74]. The *C. burnetii* genome does not appear to contain *sufA* (or its homologue *iscA*) or *sufE*, which is known to increase the activity of *csdB* 50-fold [72].

Omsland *et al*. previously reported that *C. burnetii* was able to oxidize proline under microaerobic conditions [4]. Bacteria utilize proline for protein synthesis [75, 76] and for protection against osmotic stress [77]. *C. burnetii* has been shown to actively transport glucose, glutamate and proline from the host cell intracellular environment in a pH-dependent manner [78, 79], and early work suggested that proline could be important for survival in the acidic CCV [78]. Later studies showed that *C. burnetii* uses a PmrA/B two-component system to sense the lysosomal environment and induce a transcriptional shift that mediates virulence through up-regulation of the type IV secretion system (T4SS) and associated effector proteins [80, 81], thus driving the formation of the CCV. A study by Newton *et al.* [82] has shown that the presence of proline (as well as phenylalanine and serine) is sensed by PmrB, and induces the expression of PmrA-regulated genes, subsequently inducing virulence of *C. burnetii.* Genes involved both in proline synthesis (*arcB)* and metabolism (*putA)* were essential in this study. *arcB* encodes ornithine cyclodeaminase, an enzyme capable of synthesizing proline in a single step through the deamination of ornithine [83–85]. *arcB* did not have hits against the database of essential genes, and to date, the role of *arcB* in *C. burnetii* has not been investigated. *putA* encodes a bi-functional enzyme with both proline dehydrogenase and Δ^1^-pyrroline-5-carboxylate dehydrogenase activities responsible for the metabolism of proline to glutamate. *sodB* and *sodC* encode superoxide dismutase enzymes known to protect bacteria from reactive oxygen species such as superoxide and hydrogen peroxide and have been identified as a potentially important virulence factor for *C. burnetii* [86], and have been identified as essential genes in *Burkholderia pseudomallei* [87]*.* The finding of these genes as essential in this study underpin the importance of superoxide dismutase enzymes in the protection of *C. burnetii* in the harsh intracellular environment.

### Essential genes on the QpH1 plasmid

Six predicted essential genes (three hypothetical proteins of unknown function, and the CBUA0037–39 gene cluster) were identified on the QpH1 plasmid as essential (Table 2). However, a recent report demonstrated curing *C. burnetii* NMII of this plasmid [88]. CBUA0037, CBUA0038 and CBUA0039 confer homologues of ParA, RepB and RepA – a putative origin of replication. However, these genes were still present in the QpH1-deficient strain reported by Luo *et al.* because they had been cloned into the plasmid transformed into *C. burnetii* which then triggered the loss of QpH1 [88]. Our finding that CBUA007, 0023 and 0033 are essential whilst elimination of these genes was possible in the plasmid-cured strain suggests that there are interactions between these gene products and other plasmid gene products which would otherwise be toxic.

**Table 2.** Essential genes identified in this study that are found on the QpH1 plamid

Locus tag	Gene name	Function
CBUA0007	CBUA0007	Hypothetical protein
CBUA0023	CBUA0023	Hypothetical protein
CBUA0033	CBUA0033	Hypothetical protein
CBUA0037	*parA*	Plasmid partition protein A
CBUA0038	*repB*	DNA-binding protein
CBUA0039	*repA*	Plasmid replication initiation protein

Alternatively, it is possible that these genes are not truly essential. This possibility might be addressed by generating additional transposon mutants to increase the size of the library or by the development of methods to prove essential genes in *C. burnetii *, for example using regulatable promoters to drive these genes and complementing these disrupted genes.

### Some predicted T4SS effectors are essential

None of the core components of the T4SS were classified as essential, which is to be expected as this system is generally considered to be virulence associated, with transposon mutants in almost all of these genes having been made previously [22]. However, 12 predicted T4SS effector proteins were classified as essential, ten of which are designated to encode hypothetical proteins of unknown function (Table 3). A search against the Pfam database using HMMER [89] did not reveal any significant hits for the proteins encoded by these genes, and their function remains elusive. The majority of these are predicted to be effectors based on bioinformatics approaches, looking at PmrA regulatory elements, homology to *Legionella pneumophilia* T4SS substrates, and the presence of E-blocks. Some have experimental data in the form of CyaA translocation reporter assays, but for the majority it is possible that they are not effectors after all. Establishing the biological roles of these putative effectors should be a priority for future research. For several of the potential effector genes classified as essential, neighbouring genes have also been identified as essential (see File S2). Polar effects may be influencing the classification of these genes as essential, and further studies may be required to confirm essentiality. This could be achieved through the generation of further transposon mutants to increase saturation of the pool or by complementing the transposon mutants with wild-type genes expressed from regulatable promoters.

**Table 3.** Predicted T4SS effector genes identified as essential genes in this study

Locus tag	Gene name	Function	Reference
CBU_0773	*phnB*	PhnB	[33]
CBU_0794	CBU_0794	Hypothetical protein	[90]
CBU_0881	CBU_0881	Hypothetical cytosolic protein	[90]
CBU_1213	CBU_1213	Ankyrin repeat protein	[91]
CBU_1314	CBU_1314	Hypothetical cytosolic protein	[90]
CBU_1349	CBU_1349	Hypothetical cytosolic protein	[90]
CBU_1370	CBU_1370	Hypothetical membrane associated protein	[92]
CBU_1607	CBU_1607	Hypothetical protein	[33]
CBU_1614	CBU_1614	Hypothetical protein	[81]
CBU_1686	CBU_1686	Hypothetical protein	[81]
CBU_1819	CBU_1819	Hypothetical membrane associated protein	[93]
CBUA0023	CBUA0023	Hypothetical protein	[94]

### Conclusion

We present the first report of a whole genome TraDIS study in *C. burnetii *. Data analysis using the Whiteley lab pipeline revealed 511 genes essential for the growth and survival of *C. burnetii* NMII. It was noted that some of the essential genes identified, such as predicted T4SS effectors, are typically considered to be virulence-associated. This may be a consequence of the use of ACCM-2 media to grow the bacteria, which mimics the acidic *Coxiella-*containing vacuole that *C. burnetii* would reside in, within host cells. Essential routes of synthesis were identified for the mevalonate pathway, as well as peptidoglycan and biotin synthesis. In addition, further evidence of the requirement of l-cysteine and proline for axenic growth was shown. To date, methodologies to prove essential genes in *C. burnetii* have not been developed. This should be considered a matter of priority. In other species, this has been achieved by putting essential genes under the control of an inducible promoter. In addition, it is important to note that *C. burnetii* NMII is a derogated surrogate for the fully virulent *C. burnetii* NMI, and therefore it would be valuable to carry out a TraDIS screen in this more clinically relevant strain. Nonetheless we have ongoing work evaluating the suitability of these essential genes as possible novel drug targets.

## Supplementary Data

Supplementary material 1

Supplementary material 2
