# Supplementary material for: Identification of essential genes in Coxiella burnetii
Source: Microb Genom. 2023 Feb 1;9(2):mgen000944. doi: 10.1099/mgen.0.000944 (PMC9997736; doi:10.1099/mgen.0.000944)
Supplement: Supplementary material 1 [file mgen-9-944-s001.pdf]

Supplementary table 1. Adaptors and primers used in this study. The barcode index of each multiplex primer (MPX) is underlined. Asterisks indicate a phosphorothioate modification. PCR amplification was performed using Himar-PCR-3 and one of the MPX primers. Sequencing was performed using Himar-seq2 and an adaptor-specific primer.

**Primer**	**Sequence**
**Adaptor-1**	5'-GATCGGAAGAGCACACGTC*T
**Adaptor-PCR-1**	5'-GTGACTGGAGTTCAGACGTGTGCTCTTCCGATC*T
**Himar-PCR-3**	5'-AATGATACGGCGACCACCGAGATCTACACAGTCAGTTATTGGTACCCTTAAAC*G
**MPX1**	5'-CAAGCAGAAGACGGCATACGAGATATCACGGTGACTGGAGTT*
**MPX2**	5'-CAAGCAGAAGACGGCATACGAGATCGATGTGTGACT<br>GGAGTT*C
**MPX3**	5'-CAAGCAGAAGACGGCATACGAGATTTAGGCGTGACT<br>GGAGTT*C
**Himar-seq2**	5'-CAGACCGGGGACTTATCAGCCAACC
